# Naringenin in pancreatic cancer: emerging evidence on the tumor immune-stromal microenvironment, immunometabolic reprogramming, microbiota-host crosstalk, and therapeutic resistance

**DOI:** 10.3389/fimmu.2026.1955015

**Published:** 2026-10-20

**Authors:** Yuqi Zhou

**Affiliations:** Key Laboratory for Molecular Enzymology of the Ministry of Education, School of Life Sciences, Jilin University, Changchun, China

**Keywords:** immunometabolic reprogramming, microbiota–host crosstalk, naringenin, pancreatic cancer, tumor immune–stromal microenvironment

## Abstract

Pancreatic cancer continues to be among the deadliest malignancies because of its aggressive biology, dense stromal structure, profound immunosuppression, and restricted responsiveness to existing treatment approaches. Accumulating evidence indicates that effective intervention likely depends on coordinated regulation of the tumor immune–stromal microenvironment, metabolic plasticity, and host–tumor interactions, rather than on malignant-cell targeting alone. Naringenin, a naturally occurring citrus flavanone, has received increasing attention due to its wide-ranging biological effects, including anti-inflammatory, antioxidant, immunomodulatory, and metabolic regulatory activities. This narrative review critically evaluates the current evidence on the molecular mechanisms through which naringenin affects pancreatic cancer progression, with a particular focus on tumor immune–stromal interactions, immunometabolic reprogramming, microbiota–host signaling, and therapeutic resistance. We examine its effects on oncogenic signaling pathways, immune cell function, cancer-associated fibroblasts, extracellular matrix remodeling, oxidative stress, mitochondrial homeostasis, and regulated cell death, while emphasizing growing evidence that connects dietary polyphenols with gut microbiota-derived metabolites and systemic immune regulation. The review also assesses the potential of naringenin as an adjuvant to conventional chemotherapy, targeted therapy, and immunotherapy, while recognizing the constraints arising from low bioavailability, heterogeneous preclinical models, and limited clinical evidence. Finally, we highlight key knowledge gaps and outline future research priorities, including biomarker-guided patient stratification, advanced drug delivery systems, and translational studies that combine multi-omics technologies with mechanistic investigations. Improved understanding of these interrelated mechanisms may support the rational development of naringenin-based therapeutic strategies for pancreatic cancer.

## 1 Introduction

Pancreatic cancer continues to represent one of the most difficult malignancies in contemporary oncology, accompanied by steadily rising global incidence and mortality. Based on worldwide cancer statistics, pancreatic cancer is a major contributor to cancer-related deaths globally, largely due to delayed diagnosis, rapid and aggressive disease progression, and poor overall therapeutic effectiveness (1). Pancreatic ductal adenocarcinoma (PDAC) makes up most pancreatic cancer cases and is defined by early metastatic spread, extensive genetic heterogeneity, and marked refractoriness to currently available treatments (2). Although advances have been made in surgical care, chemotherapy protocols, and molecular profiling, long-term survival remains low, underscoring the pressing need for additional treatment approaches that can target the complex biology underlying this disease (3). The treatment challenge posed by pancreatic cancer is closely linked to its distinctive tumor microenvironment, characterized by evolving interactions among malignant epithelial cells, cancer-associated fibroblasts (CAFs), immune cell populations, extracellular matrix constituents, vascular structures, and metabolic networks. A defining feature of PDAC is its dense desmoplastic stroma, which makes up a large share of the tumor mass and drives changes in drug delivery, immune exclusion, and tumor progression (4). Activated fibroblasts in the pancreatic tumor microenvironment can control extracellular matrix remodeling, inflammatory signaling, and cancer cell survival, thereby shaping disease development and the degree of response to therapy (5). At the same time, pancreatic tumors are characterized by highly immunosuppressive milieus, marked by reduced antitumor immune function, build-up of myeloid populations that promote tumor growth, and the impaired activity of cytotoxic lymphocytes (6). Together, these results indicate that pancreatic cancer progression depends not only on intrinsic abnormalities of tumor cells but also on intricate interactions within the surrounding biological environment. Another key hallmark of pancreatic cancer biology is metabolic adaptation. Tumor cells carry out extensive metabolic reprogramming to preserve proliferation and survival in the setting of nutrient deprivation, hypoxia, and oxidative stress. Alterations in glucose metabolism, mitochondrial function, amino acid utilization, and redox homeostasis contribute to tumor aggressiveness and may influence responses to anticancer therapies (7).

Moreover, accumulating evidence indicates that metabolic pathways are tightly linked to immune control, giving rise to an immunometabolic framework that affects tumor development and treatment outcomes. Consequently, therapeutic strategies that can concurrently modulate inflammatory signaling, metabolism, immune functions, and stromal interactions have attracted growing interest. Within this setting, dietary flavonoids have been identified as promising bioactive compounds, with potential importance for cancer prevention and as adjuncts to supportive therapeutic regimens. Flavonoids are naturally occurring polyphenols that are abundant in fruits, vegetables, and plant-based foods, and they have been studied due to their antioxidant, anti-inflammatory, and regulatory actions across multiple cellular pathways (8). Experimental research further implies that flavonoids may impact cancer-relevant mechanisms, such as cellular proliferation, apoptosis, angiogenesis, oxidative stress responses, immune regulation, and metabolic signaling (9). Nonetheless, their translation to clinical use is still difficult because of variability in bioavailability, metabolism, tissue distribution, and the extent to which current support is drawn from preclinical models. For this reason, mechanistic interpretation and careful assessment of translational relevance are necessary. Among dietary flavonoids, naringenin (a citrus-derived flavanone found mainly in grapefruit and related citrus fruits) has attracted substantial interest due to its wide-ranging biological activities. Prior studies have indicated that naringenin can influence inflammatory pathways, oxidative stress responses, cellular proliferation, and metabolic regulation across multiple disease models (10). Experimental research further points to potential anticancer effects of naringenin, mediated through the control of apoptosis-related processes, cell cycle progression, redox homeostasis, and signaling networks that support tumor survival (11). However, direct evidence regarding pancreatic cancer is still relatively scarce, and most existing data come from cellular and animal models rather than clinical studies. In addition to effects on tumor cells themselves, more recent work has broadened the proposed biological significance of naringenin to encompass larger regulatory systems involving immunity, metabolism, and host–microbiota interactions. Dietary polyphenols can be transformed by gut microbes, producing metabolites that may affect systemic inflammation, immune responses, and metabolic homeostasis (12).

Increasingly, the gut microbiota has been identified as a key regulator of cancer biology and treatment response; nonetheless, the precise role of naringenin-driven microbiota modulation in pancreatic cancer is still not fully clarified (13). In addition, flavonoids have been examined for their capacity to modulate immune-associated pathways and inflammatory mediators, indicating potential crosstalk with the tumor immune microenvironment (14). Contemporary progress in cancer biology has also highlighted mitochondrial quality control, ferroptosis, autophagy, and immunogenic cell death as possible therapeutic vulnerabilities in pancreatic cancer (15). Naringenin has been reported to impact several of these cellular processes across different experimental settings, but it remains to be determined whether such mechanisms can be translated into effective pancreatic cancer therapy (16). Moreover, advancing bioavailability through nanotechnology-based delivery systems and combination strategies constitutes an important future avenue for strengthening the therapeutic promise of naturally derived compounds (17).

Accordingly, this narrative review seeks to critically appraise the emerging role of naringenin in pancreatic cancer by synthesizing evidence on tumor immune–stromal microenvironment remodeling, immunometabolic control, microbiota–host signaling, and resistance to therapy. We examine current mechanistic findings, translational constraints, and key future research directions to determine whether naringenin might support the development of more personalized, biologically grounded treatment strategies for pancreatic cancer.

## 2 Methods

### 2.1 Literature search strategy

This narrative review aimed to determine and critically integrate the existing evidence on the biological mechanisms, therapeutic targets, and translational potential of naringenin in pancreatic cancer. A structured literature search strategy was used to enhance transparency and reproducibility while preserving the flexibility inherent to a narrative review. The search covered major biomedical databases, including PubMed/MEDLINE, Scopus, the Web of Science Core Collection, and Google Scholar. Searches were run from each database’s inception through May 2026. The search approach was designed to include studies examining how naringenin affects pancreatic cancer biology, encompassing tumor cell signaling, immune–stromal interactions, immunometabolic regulation, microbiota–host communication, and therapeutic responses. Throughout the process, the search was directed by central concepts tied to the bioactive compound under investigation, pancreatic cancer–related conditions, and molecular, cellular, and translational outcomes. Additional manual searches were conducted of the reference lists from pertinent articles and recent reviews to find potentially eligible studies that had not been captured by the electronic database searches. Priority was assigned to original research articles, mechanistic studies, translational investigations, and high-quality reviews offering conceptual or biological insights aligned with the aims of this review.

### 2.2 Databases and search terms

The literature review was conducted using PubMed/MEDLINE, Scopus, the Web of Science Core Collection, and Google Scholar. To maximize the retrieval of pertinent studies, a combination of controlled vocabulary terms and free-text keywords was used. The search approach involved combinations of terms related to naringenin, pancreatic cancer, tumor biology, immune regulation, metabolism, microbiota, and therapeutic response. The principal search string was organized as follows: (“naringenin” OR “naringenin supplementation” OR “citrus flavanone” OR “flavonoid”) AND (“pancreatic cancer” OR “pancreatic carcinoma” OR “pancreatic ductal adenocarcinoma” OR “PDAC”) AND (“tumor microenvironment” OR “immune microenvironment” OR “immunometabolism” OR “inflammation” OR “oxidative stress” OR “mitochondria” OR “apoptosis” OR “autophagy” OR “ferroptosis” OR “metabolism” OR “drug resistance” OR “chemotherapy” OR “immunotherapy” OR “microbiota” OR “gut microbiome” OR “host–microbiota interaction”). Additional targeted searches were performed using combinations of naringenin with specific molecular and translational terms, including KRAS signaling, the phosphoinositide 3-kinase (PI3K)/protein kinase B (Akt) pathway, nuclear factor kappa B cells (NF-κB) activation and AMP-activated protein kinase/mammalian target of rapamycin (AMPK/mTOR) regulation, CAFs, immune modulation, and nanoparticle-based delivery strategies. The search outputs were first screened for relevance by title and abstract, followed by full-text review of studies that appeared potentially eligible. While no restriction on publication year was imposed, greater priority was given to more recent studies that offer mechanistic, translational, and clinically relevant insights.

### 2.3 Eligibility criteria

Studies were included for eligibility when they centered on naringenin as the primary bioactive compound and evaluated its biological or therapeutic significance in contexts related to pancreatic cancer. Original experimental studies were deemed relevant if they employed pancreatic cancer cell models, animal models, patient-derived models, or translational approaches and examined mechanisms associated with tumor progression, cell survival, inflammatory signaling, oxidative stress, mitochondrial regulation, metabolic reprogramming, immune modulation, remodeling of the tumor microenvironment, microbiota-associated mechanisms, or therapeutic sensitivity. Relevant review articles were also included when they provided key conceptual frameworks or synthesized newly emerging mechanisms concerning naringenin biology and pancreatic cancer. Studies were excluded if they did not directly address pancreatic cancer, did not include a specific focus on naringenin, or examined complex mixtures of dietary compounds without permitting the independent contribution of naringenin to be determined. Conference abstracts, editorials, non-peer-reviewed publications, and articles lacking accessible full-text information were also excluded. Because this study was structured as a narrative review, studies were not chosen for quantitative synthesis, and no meta-analysis was conducted. Findings obtained from indirect models or from non-pancreatic cancer systems were interpreted with caution and treated mainly as supportive mechanistic evidence rather than as conclusive proof of therapeutic efficacy.

### 2.4 Study selection

The study selection process followed a series of sequential screening steps. At the outset, the retrieved records were screened by title and abstract to identify publications that were potentially relevant. Duplicate records found across databases were removed prior to screening. Articles judged to be potentially eligible were then assessed through full-text review in accordance with the predefined inclusion and exclusion criteria. During the full-text evaluation, studies were chosen based on how directly they addressed the core topics of this review: remodeling of the tumor immune–stromal microenvironment, immunometabolic regulation, microbiota–host interactions, and therapeutic resistance in pancreatic cancer. Greater emphasis was placed on studies that offered mechanistic explanations rather than purely descriptive observations. If the evidence was obtained from non-pancreatic cancer models or from indirect biological settings, the findings were interpreted with caution and presented as hypothesis-generating, rather than as conclusive evidence, for pancreatic cancer therapeutic approaches.

### 2.5 Data extraction and synthesis

Relevant information reported in the included studies was extracted and arranged according to study characteristics, experimental model, intervention parameters, examined molecular pathways, key biological outcomes, and translational implications. The extracted data comprised the study, the pancreatic cancer model, the available naringenin formulation and concentration, the mechanisms evaluated, the signaling pathways affected, and the reported effects on tumor biology or therapeutic response. The gathered evidence was then narratively synthesized within a mechanistic framework rather than presented as a study-by-study account. Results were consolidated into major conceptual areas, including direct effects of naringenin on pancreatic cancer cells, regulation of the tumor immune–stromal microenvironment, immunometabolic control, microbiota–host interactions, and possible contributions to modulating therapeutic resistance. In addition, contradictory findings, limitations of the experimental models, differences in dosing strategies, and challenges related to bioavailability and clinical translation were taken into account. Overall, the interpretation highlighted the difference between well-established biological effects and emerging hypotheses that still require further validation in clinically relevant models.

## 3 Pharmacological characteristics of naringenin relevant to pancreatic cancer

### 3.1 Chemical structure and natural dietary sources

Naringenin is a naturally occurring flavanone within the flavonoid family of plant-derived polyphenols. From a structural standpoint, it features the typical C6–C3–C6 flavonoid scaffold, made up of two aromatic rings (A and B rings) linked via a heterocyclic C ring, and it bears hydroxyl substitutions that shape both its biological activity and chemical characteristics (9). In foods, naringenin is often found as glycosylated compounds, especially naringin, which is abundant in citrus fruits including grapefruit, oranges, and mandarins. After consumption, naringin is hydrolyzed by intestinal enzymes and β-glucosidases produced by the gut microbiota to yield the aglycone naringenin form, which is regarded as the biologically active metabolite (18). The structural properties of naringenin determine its interactions with cellular proteins, its antioxidant behavior, and its metabolic conversion. Specifically, the hydroxyl groups present in its flavonoid framework support electron-donating ability and may enable interactions with signaling molecules, although these same chemical features also affect its solubility and absorption profile. Despite the extensive investigation of naringenin owing to its wide-ranging biological activities, including anti-inflammatory and metabolic regulatory effects, its clinical use is still constrained by pharmacokinetic limitations (12). From a nutritional standpoint, citrus-derived flavonoids constitute a significant dietary source of exposure to naringenin. Epidemiological research has indicated that greater intake of flavonoid-rich foods is associated with beneficial health outcomes; however, drawing direct conclusions about cancer prevention or therapy remains challenging because dietary patterns include multiple bioactive compounds and are shaped by a wide range of lifestyle influences (18). Consequently, with respect to pancreatic cancer, naringenin should presently be regarded as a bioactive molecule with therapeutic promise that calls for additional pharmacological refinement, rather than as a validated dietary anticancer strategy.

### 3.2 Absorption, metabolism, and bioavailability

Although naringenin shows promising biological activities, its therapeutic development is strongly shaped by its low oral bioavailability. After dietary consumption, naringenin is taken up mainly in the form of glycosylated derivatives, such as naringin, rather than as the free aglycone. In the gastrointestinal tract, these glycosides are cleaved by enzymatic hydrolysis, producing naringenin that can then be absorbed via intestinal epithelial pathways (19). Nevertheless, pronounced first-pass metabolism markedly affects circulating levels and biological availability. Once absorbed, naringenin is subjected to phase II metabolic processes, including glucuronidation and sulfation, which are primarily driven by UDP-glucuronosyltransferases and sulfotransferases. As a result, circulating naringenin is found largely in conjugated forms instead of as the free aglycone (20). These metabolites may exhibit biological properties distinct from those of the parent compound, yet their specific role in anticancer activity is still not fully understood. The gut microbiota substantially contributes to the metabolism of naringenin. Microbial enzymes are able to convert flavonoids into smaller phenolic metabolites, which may modulate systemic biological effects. This interaction underscores the need to view naringenin not only as a standalone compound, but also as a component of an intricate host–microbiota metabolic network (21). Nevertheless, differences in gut microbial composition, dietary habits, and individual metabolic capacity may account for variability in naringenin exposure across people. In pancreatic cancer contexts, limited bioavailability is among the main translational obstacles. While *in vitro* experiments frequently utilize naringenin, there is a substantial gap between effective *in vitro* concentrations and achievable *in vivo* plasma levels. Preclinical studies evaluating naringenin’s efficacy in PDAC cell lines, such as PANC-1, AsPC-1, and SUIT-2, typically require concentrations ranging from 50 µM to 300 µM to successfully suppress migration, alter metabolic markers, or induce apoptosis. Conversely, human pharmacokinetic data demonstrates that a single oral dose of 135 mg of pure naringenin yields a peak plasma concentration (Cmax) of only about 7.4 µM (approximately 2000 ng/mL). This significant discrepancy between the high micromolar concentrations required for direct tumoricidal activity in cell culture and the single-digit micromolar concentrations attainable in human systemic circulation severely limits standard oral monotherapy. Consequently, advanced delivery systems, such as nanoparticle encapsulation, co-crystal formulations, or targeted adjuvant approaches, are absolutely essential to bridge this translational gap and achieve therapeutic concentrations within the pancreatic tumor microenvironment (22, 23).

### 3.3 Pharmacokinetics and tissue distribution

The pharmacokinetic profile of naringenin involves quick absorption followed by widespread metabolism and excretion. After oral dosing, circulating levels of unbound naringenin are typically low due to conjugation occurring in the intestine and liver. Pharmacokinetic research has shown that glucuronidated and sulfated metabolites constitute the main circulating species, indicating that overall systemic exposure is driven primarily by the generation and clearance of metabolites rather than by unbound naringenin concentrations alone (24). Studies on distribution suggest that naringenin metabolites may access multiple tissues, though their levels differ substantially with the route of administration, formulation, dose, and metabolic condition. Experimental findings have identified flavonoid metabolites in tissues participating in metabolic regulation, implying that naringenin-derived compounds could produce biological effects beyond the gastrointestinal tract (25). Nevertheless, direct evidence supporting accumulation in pancreatic tissue or in pancreatic tumors is still scarce. The link between plasma pharmacokinetics and biological activity at the tissue level continues to be a crucial factor in cancer applications. Many anticancer effects attributed to naringenin stem from cellular studies conducted at concentrations that may be higher than what is attainable through systemic exposure (26). Consequently, these results must be interpreted with caution, and pharmacologically relevant models are needed to assess whether adequate concentrations can be reached at pancreatic tumor sites. Advancing knowledge of naringenin pharmacokinetics could support the rational design of combination strategies and improved formulations. Monitoring circulating metabolites, assessing tissue distribution, and combining these data with pharmacodynamic analyses will be essential to determine whether naringenin can produce biologically significant exposure in pancreatic cancer contexts.

### 3.4 Nanoformulations and drug-delivery strategies

The combination of limited water solubility, extensive metabolism, and comparatively low systemic exposure has driven the creation of advanced delivery systems aimed at improving naringenin bioavailability. Nanotechnology-based strategies such as polymeric nanoparticles, lipid-based carriers, nanoemulsions, and other encapsulation platforms, have been explored to increase solubility, shield the compound from degradation, and support controlled release (27). In the context of cancer research, nanoformulations may offer further benefits by enhancing pharmacokinetic characteristics and enabling more effective transport of bioactive compounds to tumor tissues. Studies using naringenin-loaded nanoparticles have reported greater cellular uptake and improved biological activity relative to free naringenin in some experimental models (28). Nevertheless, the majority of current findings are still limited to preclinical work, and it remains to be determined whether these delivery systems can yield clinically relevant advantages in pancreatic cancer through further investigation. Several challenges still need to be addressed before naringenin-based nanomedicines can be implemented in clinical settings. Key priorities include improving formulation stability, enabling large-scale manufacturing, conducting safety evaluations, assessing biodistribution, and addressing regulatory requirements (29). Moreover, nanoparticle systems should be tested not only for enhanced drug exposure but also for their capacity to preserve the biological properties of naringenin metabolites. Taken together, sophisticated delivery approaches offer a promising path for mitigating pharmacological constraints linked to naringenin. Future research that combines pharmacokinetics, tumor-targeted delivery, and clinically relevant pancreatic cancer models may help clarify whether optimized naringenin formulations can support therapeutic development (**Table 1**).

**Table 1:** Pharmacological characteristics of naringenin relevant to pancreatic cancer therapy.

Category	Characteristics	Potential relevance to pancreatic cancer	Advantages	Limitations
Natural sources	Mainly derived from citrus fruits; commonly present as naringin glycoside	Provides dietary origin and biological accessibility	Naturally occurring, widely available	Dietary intake may not achieve pharmacological concentrations
Chemical properties	Citrus flavanone with hydroxylated flavonoid structure	Determines biological interactions and antioxidant capacity	Multi-target molecular activity	Limited solubility
Bioavailability	Low oral bioavailability due to poor absorption and extensive metabolism	Major barrier for therapeutic translation	Can be improved through formulation strategies	Variable individual exposure
Metabolism	Converted into glucuronidated, sulfated, and microbial-derived metabolites	Metabolites may contribute to biological effects	Generates bioactive derivatives	Metabolic variability
Major metabolites	Naringenin glucuronides, sulfates, and microbial phenolic metabolites	May influence systemic responses	Potential pharmacological activity	Biological roles remain incompletely defined
Nanoformulations	Nanoparticles, nanoemulsions, lipid-based delivery systems	May enhance tumor exposure and stability	Improved solubility and delivery	Mostly preclinical evidence
Overall advantages	Broad biological activities and multi-pathway regulation	Supports investigation as an adjunctive strategy	Mechanistically diverse effects	No established clinical efficacy
Overall limitations	Pharmacokinetic constraints and limited human evidence	Requires further translational studies	Amenable to optimization	Clinical validation lacking

## 4 Naringenin and tumor cell-intrinsic mechanisms in pancreatic cancer

### 4.1 Regulation of oncogenic signaling networks

The onset and progression of pancreatic cancer are strongly shaped by the persistent activation of oncogenic signaling networks that control proliferation, survival, differentiation, and cellular adaptation. Among these changes, activating KRAS mutations are among the earliest and most common molecular alterations in PDAC, driving continued activation of downstream pathways such as rapidly accelerated fibrosarcoma–mitogen-activated protein kinase kinase–extracellular signal-regulated kinase and PI3K–Akt signaling pathways signaling (30). Since direct pharmacological inhibition of mutant KRAS has long been difficult, research attention has increasingly shifted toward compounds that can modulate multiple signaling targets required for tumor cell maintenance (31). Naringenin, a citrus-derived flavanone, has been examined as a candidate modulator of intracellular signaling pathways implicated in malignant cellular behavior.

Direct evidence evaluating naringenin on oncogenic signaling in pancreatic cancer remains limited. Studies in PDAC cell lines demonstrate that naringenin suppresses cell viability and migratory signaling, but much of the granular pathway mapping (such as PI3K/Akt and MAPK inhibition) stems from non-pancreatic cancer models (11). These non-PDAC findings provide a helpful biological rationale, yet whether naringenin achieves similar pathway selectivity against KRAS-driven signaling in primary pancreatic tumors requires dedicated investigation.

Given the ubiquitous nature of KRAS mutations in PDAC, targeting its downstream effector cascades, namely the PI3K/Akt and MAPK/ERK pathways, is of critical therapeutic importance (32, 33). Emerging studies have demonstrated that naringenin directly interferes with these oncogenic networks in pancreatic cancer models. Specifically, naringenin has been shown to downregulate the phosphorylation of AKT and mTOR, effectively blunting the PI3K/AKT/mTOR survival axis and inducing apoptosis in PDAC cell lines such as PANC-1 and AsPC-1 (23). Furthermore, naringenin exhibits targeted inhibitory effects on the MAPK pathway by reducing the activation of ERK1/2, thereby suppressing tumor cell proliferation, migration, and metastatic potential (11, 34). By dual-targeting these critical KRAS-driven downstream networks, naringenin actively counteracts the primary oncogenic drivers of PDAC progression, providing a strong biological rationale for its role in disrupting tumor signaling (16).

Prior studies have proposed that naringenin could modulate these pathways by lowering the activation of inflammatory mediators in experimental systems (12). Likewise, signaling networks involving TGF-β and Wnt/β-catenin contribute to epithelial plasticity and tumor progression, although the precise effects of naringenin on these pathways in pancreatic cancer have not been fully defined. Consequently, the available evidence is consistent with the notion that naringenin may influence pancreatic cancer-associated signaling networks through coordinated regulation across multiple pathways, rather than functioning as a single-target inhibitor. Additional mechanistic investigations are needed to establish whether these effects result in a biologically significant suppression of PDAC progression.

### 4.2 Immunometabolic reprogramming

Metabolic reprogramming is a defining feature of pancreatic cancer cells, allowing them to adjust to changes in nutrient supply and to sustain ongoing proliferation. In PDAC, cells often show increased glycolytic activity, modified mitochondrial metabolism, and a higher biosynthetic capacity; together, these traits promote tumor cell survival in stressful environments (7). While metabolic changes are closely connected to immune and stromal interactions within the tumor microenvironment, this section concentrates specifically on the direct impacts of naringenin on tumor cell metabolic control. Naringenin has been investigated as a potential metabolic modulator due to its reported effects on glucose metabolism, lipid homeostasis, and regulation of cellular energy. Findings from metabolic disorder and cancer models indicate that naringenin may affect glucose utilization and pathways related to glycolysis, though its exact effects seem to vary with cellular context and the experimental conditions (35). Because pancreatic cancer cells frequently rely on dysregulated glucose metabolism, determining whether naringenin can alter glycolytic adaptation is an important topic for future research. Energy-sensing pathways especially the AMPK–mTOR axis, govern the trade-off between anabolic growth and metabolic conservation. AMPK activation typically induces energy-preserving responses, whereas mTOR signaling facilitates biosynthesis and cellular proliferation. Work across different experimental settings has indicated that naringenin may activate AMPK-associated pathways and inhibit mTOR-related signaling, which could modulate tumor cell metabolism (36).

While immunometabolic reprogramming plays a critical role in the progression and immune evasion of pancreatic ductal adenocarcinoma (PDAC), direct evidence demonstrating naringenin’s specific impact on these metabolic pathways within PDAC models remains highly limited. Currently, the capacity of naringenin to modulate key metabolic nodes, including glucose uptake, glycolytic flux, lactate production, and the expression of GLUT1, HK2, and LDHA, is primarily extrapolated from its documented effects in metabolic diseases and other cancer types. Similarly, the potential regulation of mitochondrial respiration, lipid metabolism, glutamine metabolism, and hypoxia/HIF-1α by naringenin must be viewed strictly as proposed mechanisms in PDAC rather than demonstrated effects. For example, while naringenin has been hypothesized to activate AMPK and inhibit mTOR signaling based on general metabolic principles (37) and evidence from non-PDAC models, dedicated PDAC-specific investigations are strictly required to validate these immunometabolic targets and confirm whether naringenin actively drives these metabolic shifts in pancreatic cancer cells.

Furthermore, HIF-1α–driven metabolic adaptation contributes substantially to hypoxic tumor microenvironments by enhancing glycolysis and supporting survival. Naringenin has been reported to affect hypoxia-related responses in some cancer models; however, direct evidence in pancreatic cancer is still lacking. Changes in lipid metabolism are also increasingly viewed as key elements of PDAC biology. Although preliminary studies suggest that naringenin may regulate lipid-related pathways, whether these effects contribute directly to pancreatic cancer suppression remains unclear. Thus, current findings provide a mechanistic rationale for investigating naringenin as a potential metabolic regulator, but additional PDAC-specific studies are required.

Beyond tumor-intrinsic metabolism, the immunometabolic regulatory capacity of naringenin extends to the metabolic reprogramming of surrounding immune cells, which is a critical factor in remodeling the tumor microenvironment. In macrophages, naringenin has been shown to suppress glycolysis and promote lipid oxidation, thereby shifting the metabolic phenotype away from a pro-inflammatory M1 state toward an anti-inflammatory M2 state. This metabolic rewiring is mechanistically linked to the inhibition of the NLRP3 inflammasome and enhanced cholesterol efflux. Moreover, naringenin directly influences T-cell metabolic fitness. It acts as a natural immunomodulator by translating extracellular metabolic signals into altered gene expression, notably inducing regulatory T-cell differentiation through an aryl hydrocarbon receptor mediated pathway. Although these immunometabolic effects have been primarily characterized in inflammatory and autoimmune models, they highlight a dual metabolic mechanism where naringenin simultaneously disrupts tumor energy pathways and reshapes immune cell metabolic polarization. Validating these specific macrophage and T-cell metabolic shifts in PDAC models remains an essential next step ref.

### 4.3 Oxidative stress, mitochondrial homeostasis, and regulated cell death

Pancreatic cancer cells are subjected to substantial oxidative burden as a result of intense proliferative activity, mitochondrial dysfunction, and metabolic stress. To preserve survival, tumor cells control reactive oxygen species (ROS), mitochondrial quality control, and several categories of regulated cell death. These processes are key determinants of tumor cell survival and may offer targets for therapeutic development. Naringenin has long been described as an antioxidant flavonoid; nevertheless, its biological actions likely reflect more intricate modulation of redox signaling than straightforward elimination of ROS. Experimental evidence suggests that naringenin can regulate intracellular ROS concentrations and modulate antioxidant defense pathways, thereby reshaping cellular responses to oxidative stress (38). In cancer cells, a regulated increase in ROS may promote activation of apoptotic cascades, while overly strong ROS inhibition could disrupt normal signaling functions. Mitochondrial dysfunction is another key characteristic of pancreatic cancer biology. Naringenin has been reported to modulate mitochondrial membrane potential, mitochondrial enzyme activity, and pathways related to mitochondrial integrity across various experimental models (39). Nonetheless, the significance of these effects for PDAC cells has yet to be clearly determined. Mitophagy serves as an additional regulatory link between mitochondrial quality control and tumor survival. Naringenin has also been linked to the regulation of programmed cell death pathways, including apoptosis and autophagy. Naringenin has been reported to modulate mitochondrial membrane potential and enhance apoptosis through the generation of reactive oxygen species specifically in human pancreatic cancer cells (40). Furthermore, flavonoids have been evaluated for possible effects on ferroptosis, an iron-dependent form of cell death defined by lipid peroxidation (41); however, evidence connecting naringenin directly to ferroptotic regulation in pancreatic cancer is still developing. Overall, available evidence suggests that naringenin may influence oxidative balance, mitochondrial function, and cell death pathways in tumor cells. Nevertheless, most findings remain preclinical, and the precise contribution of these mechanisms to pancreatic cancer therapy requires further investigation.

## 5 Naringenin-mediated immunomodulatory mechanisms relevant to pancreatic cancer

While naringenin is frequently characterized in the literature as a potent antioxidant, its biological activity exhibits a concentration-dependent duality, functioning alternately as an antioxidant or a pro-oxidant depending on the cellular microenvironment and local redox state (42). At physiological or low concentrations, naringenin exerts protective antioxidant effects primarily through direct scavenging of reactive oxygen species (ROS) and hydrogen donation from its phenolic hydroxyl groups (43). However, at elevated concentrations or in specific microenvironments, such as those with high baseline oxidative stress or high levels of transition metal catalysts like iron and copper, flavonoids can undergo auto-oxidation (44, 45). This process generates reactive intermediates that paradoxically drive the intracellular production of ROS via Fenton-type reactions. Recognizing this apparent paradox is critical for understanding naringenin’s pleiotropic effects in cancer models: while low-dose antioxidant activity typically supports cytoprotection in healthy tissues, higher-dose pro-oxidant generation can selectively trigger apoptotic pathways in pathological cells or alter vital redox-sensitive signaling cascades in the tumor microenvironment (40, 46).

### 5.1 Macrophage activation and innate immune regulation

The tumor immune microenvironment in PDAC shows profound dysregulation of myeloid cells. In this setting, macrophages often adopt immunosuppressive phenotypes that facilitate tumor advancement, extracellular matrix remodeling, and resistance to therapy. Direct experimental data examining naringenin within the PDAC immune microenvironment are currently lacking. Available insights are derived entirely from non-pancreatic systems, including oral squamous cell carcinoma and lipopolysaccharide-stimulated macrophage models (47, 48). While these non-PDAC studies indicate that naringenin can alter macrophage polarization and cytokine release, these indirect findings must be interpreted with caution given the unique desmoplastic and immunosuppressive architecture of pancreatic tumors. A recurring mechanistic theme across the available studies is that naringenin can shift macrophage activation states by suppressing excessive NF-κB–mediated inflammatory signaling. In lipopolysaccharide (LPS)-stimulated macrophages, naringenin decreased the release of pro-inflammatory mediators including tumor necrosis factor-alpha, interleukin-1 beta, and interleukin-6, by promoting metallothionein 1G and then inhibiting NF-κB activation (48). Similarly, experimental models of LPS-induced inflammatory responses showed that naringenin reduced macrophage-associated inflammatory activity, leukocyte recruitment, oxidative stress, and cytokine production by suppressing NF-κB signaling (49). Taken together, these results suggest that naringenin can modulate macrophage inflammatory polarization; however, it is still unclear whether these effects also translate into the reprogramming of tumor-associated macrophages (TAMs) toward an antitumor phenotype in PDAC. Importantly, the immunological outcomes of macrophage modulation appear to depend strongly on the biological context. In contrast to chronic inflammatory conditions, in which excessive macrophage activation can drive tissue damage, macrophages associated with cancer often display immunosuppressive and tumor-promoting features. Consequently, simply decreasing inflammatory signaling may not necessarily lead to improved antitumor immunity. The strongest evidence for a possible anticancer immune effect comes from Kawaguchi et al. (47), who showed that administering naringenin increased CD169-positive macrophages in draining lymph nodes, boosted IL-12 and CXCL10 expression, and enhanced cytotoxic T lymphocyte infiltration in murine oral squamous cell carcinoma models (47). These results indicate that naringenin may modulate macrophage functions linked to antigen presentation and T-cell recruitment, rather than merely suppressing inflammation. Still, applying these findings to PDAC requires caution. PDAC features a highly specialized desmoplastic and immunosuppressive microenvironment, which is largely composed of pancreatic stellate cells, TAMs, myeloid-derived suppressor cells, and regulatory immune populations. Variations in tumor type, macrophage origin, experimental models, and dosing approaches could markedly affect the biological response to naringenin. Consequently, the current evidence supports the hypothesis that naringenin can regulate macrophage-associated immune pathways, but direct confirmation in PDAC models remains necessary.

### 5.2 T-cell recruitment and adaptive antitumor immunity

Adaptive immune dysfunction poses a substantial obstacle to effective antitumor responses in PDAC. Relative to immunogenic tumors, PDAC typically shows restricted cytotoxic T-cell infiltration, defective antigen presentation, and several mechanisms that promote T-cell exhaustion. As a result, approaches that can bolster CD8^+^ T-cell function or strengthen immune recognition have drawn significant attention. Evidence supporting naringenin-mediated T-cell regulation is drawn exclusively from non-pancreatic tumor systems, as direct PDAC T-cell studies have not yet been reported. In hepatocellular carcinoma and oral cancer models, naringenin increased intratumoral cytotoxic T-lymphocyte infiltration and reduced checkpoint expression (47, 50). While these non-PDAC results suggest a potential for vascular and immune modulation, their applicability to the dense, immune-excluded PDAC stroma remains hypothetical until confirmed in immunocompetent pancreatic cancer models. The most compelling experimental support is provided by Kawaguchi et al. (47), in which naringenin administration in oral cancer models increased CD8^+^ T-cell infiltration into tumors, together with activation of CD169-positive macrophages and upregulated expression of immune-associated mediators such as IL-12 and CXCL10 (47). This result is consistent with a framework in which naringenin enhances immune surveillance by altering upstream innate immune elements that govern lymphocyte recruitment. Additional evidence from hepatocellular carcinoma models suggests that naringenin could modulate adaptive immunity by coordinating the regulation of angiogenic and immune checkpoint pathways. After naringenin treatment, intratumoral CD8^+^ T-cell levels increased, while PD-L1 expression decreased, mediated through VEGFA and c-Met degradation mechanisms. These results point to a possible link between vascular remodeling and improved immune accessibility (50). This line of evidence is especially pertinent to PDAC, since abnormal vasculature and immune exclusion are established factors contributing to resistance to immunotherapeutic strategies. Nevertheless, the connection between naringenin and T-cell regulation appears to be multifaceted. In non-cancer immune models, naringenin has also been shown to inhibit T-cell activation and proliferation under particular inflammatory conditions, by lowering CD69 expression, cytokine output, and signaling through activation-associated pathways (51). This apparent inconsistency underscores the context-dependent character of flavonoid-driven immune regulation. Accordingly, the impact of naringenin may vary depending on tumor-derived signals, immune cell subtype, concentration, and the length of exposure. Therefore, instead of treating naringenin as a general T-cell activator, the current body of evidence points to a more nuanced interpretation: naringenin may alter immune interactions in tumor settings by modulating the balance between immune activation and inflammatory limitation. A key translational issue is whether these effects can counteract the substantial T-cell exclusion that is typical of PDAC.

### 5.3 Angiogenic and inflammatory signaling modulation

Angiogenesis and immune regulation are increasingly understood as mutually linked processes within solid tumors. In PDAC, abnormal vascular architecture affects not only nutrient supply and tumor progression, but also immune evasion by restricting lymphocyte trafficking and enhancing immunosuppressive signaling. Consequently, agents that can concurrently modulate angiogenic pathways and immune responses may be promising candidates for combination approaches. Among the available studies, the most extensive evidence supporting naringenin-mediated control of angiogenesis and immune-related pathways is provided by Wu et al. (50). In hepatocellular carcinoma models, naringenin suppressed endothelial cell migration and tube formation, decreased neovascularization in chick chorioallantoic membrane assays, and promoted the degradation of VEGFA and c-Met via K48-linked ubiquitination mechanisms (50). Notably, these vascular effects coincided with reduced PD-L1 expression and increased CD8^+^ T-cell infiltration, indicating that naringenin may influence tumor immunity, at least in part, by reshaping the angiogenic microenvironment. The potential importance of these findings for PDAC is reinforced by the established involvement of VEGF signaling, hypovascularity, and immune exclusion in pancreatic tumors. However, PDAC differs markedly from hepatocellular carcinoma with respect to vascular biology, stromal composition, and immune organization. Consequently, the anti-angiogenic effects reported in other tumor models cannot be straightforwardly presumed to occur in pancreatic cancer. In addition to angiogenesis, naringenin consistently controls inflammatory signaling pathways, especially NF-κB-associated responses. Evidence from studies in macrophages and inflammatory models shows that naringenin can curtail excessive inflammatory cytokine production via NF-κB inhibition (48, 49). Still, deriving therapeutic conclusions from modulation of inflammation in cancer is difficult, since inflammation can function both to promote and to restrain tumor development depending on cellular context. Overall, the available evidence suggests that naringenin may affect immune-related and angiogenic pathways through coordinated regulation of macrophage activity, cytokine signaling, vascular remodeling, and immune checkpoint-linked mechanisms (**Table 2**). However, direct studies in PDAC are required to determine whether these preclinical observations can be translated into clinically meaningful improvements in immune responsiveness or treatment sensitivity (**Figure 1**).

**Table 2:** Preclinical evidence for naringenin-mediated immunomodulatory mechanisms relevant to pancreatic cancer.

Model	Immune/angiogenic target	Effects of naringenin	Evidence classification	Relevance to PDAC	Ref
Murine oral squamous cell carcinoma	CD169+ macrophages; CTL recruitment	Increased CD169+ macrophages; increased IL-12/CXCL10; increased CD8+ T cell infiltration; decreased tumor growth	Evidence in other cancer models	Suggests potential enhancement of macrophage mediated antitumor immunity and cytotoxic lymphocyte recruitment	(47)
Hepatocellular carcinoma (*in vitro*/*in vivo*); HUVEC and CAM assays	VEGFA/c-Met signaling; PD-L1; CD8+ T cells; angiogenesis	Decreased VEGFA and c-Met; decreased PD-L1; decreased angiogenesis; increased CD8+ T cells	Evidence in other cancer models	Supports potential immune vascular modulation relevant to immune exclusion in PDAC	(50)
LPS stimulated human macrophages	MT1G/NF-kB pathway; inflammatory cytokines	Increased MT1G; decreased NF-kB activation; decreased TNF-alpha, IL-1beta, IL-6 production	Evidence from non-cancer/inflammatory/metabolic models	Indicates potential regulation of macrophage inflammatory signaling within tumor associated immune networks	(48)
LPS induced mouse inflammation model; RAW264.7 macrophages	Macrophage activation; NF-kB; leukocyte recruitment	Decreased NF-kB activation; decreased cytokines; decreased macrophage/neutrophil recruitment; decreased oxidative stress	Evidence from non-cancer/inflammatory/metabolic models	Provides mechanistic evidence for modulation of inflammatory components relevant to tumor microenvironment	(49)
Contact hypersensitivity model; activated T lymphocytes	T cell activation and proliferation	Decreased T cell proliferation; decreased CD69 expression; decreased IL-2/TNF-alpha/IFN-gamma; increased apoptosis associated signaling	Evidence from non-cancer/inflammatory/metabolic models	Highlights context dependent effects of naringenin on adaptive immunity and the need for PDAC specific evaluation	(51)

CAM, chick chorioallantoic membrane; CTL, cytotoxic T lymphocyte; HUVEC, human umbilical vein endothelial cell; IL, interleukin; LPS, lipopolysaccharide; NF-κB, nuclear factor-kappa B; PDAC, pancreatic ductal adenocarcinoma; PD-L1, programmed death-ligand 1; VEGFA, vascular endothelial growth factor A.

**Figure 1:**
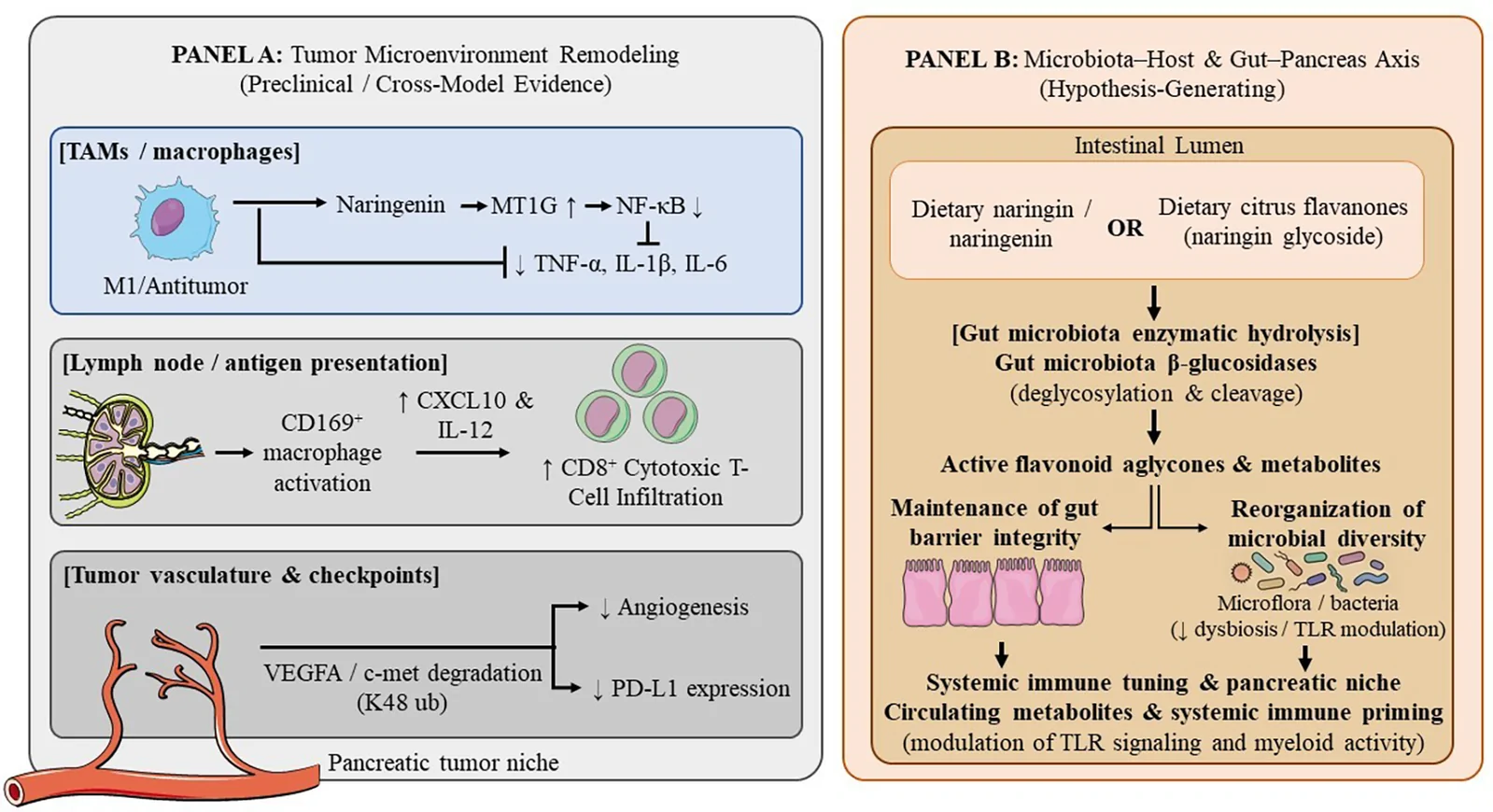
Conceptual framework of naringenin-mediated tumor immune–stromal microenvironment remodeling and host–microbiota crosstalk. **(A)** Tumor microenvironment remodeling: naringenin regulates macrophage inflammatory polarization by inducing MT1G to inhibit NF-κB activation, suppressing pro-inflammatory cytokine production (TNF-α, IL-1β, IL-6). In draining lymph nodes, naringenin stimulates CD169^+^ macrophages, upregulating CXCL10 and IL-12 to promote cytotoxic CD8^+^ T-cell infiltration into tumors. Concurrently, it drives K48-linked ubiquitination and degradation of VEGFA and c-Met, leading to reduced tumor angiogenesis and downregulation of PD-L1 expression. **(B)** Microbiota–host and gut–pancreas axis: ingested naringin/naringenin is deglycosylated and hydrolyzed by gut microbial enzymes into active flavanone aglycones and secondary phenolic catabolites. These metabolites support gut epithelial barrier integrity and reorganize intestinal microbial composition. This gut–pancreas axis crosstalk provides systemic immune tuning that influences the pancreatic tumor microenvironment. Panel **(A)** incorporates cross-model preclinical evidence; panel **(B)** represents a hypothesis-generating framework requiring clinical confirmation in human PDAC cohorts.

## 6 Naringenin and microbiota–host crosstalk

Before detailing the potential microbiota-modulating effects of naringenin, it is critical to explicitly differentiate between direct evidence in pancreatic ductal adenocarcinoma (PDAC) and evidence extrapolated from other disease contexts. Currently, direct empirical data demonstrating naringenin’s ability to therapeutically alter the gut or intratumoral microbiome specifically within a PDAC model is lacking. Consequently, the mechanisms discussed in the subsequent sections (6.1–6.3), which draw upon findings from melanoma, hepatocellular carcinoma, and non-alcoholic steatohepatitis (NASH) models, must be interpreted with caution. While these non-PDAC models provide a valuable theoretical framework for understanding naringenin’s broader microbiome-host interactions, its specific capacity to modulate the unique, highly immunosuppressive microbiome characteristic of PDAC remains speculative and requires dedicated experimental validation.

### 6.1 Gut microbiota as an immunological regulator in pancreatic cancer

The developing notion of the microbiota–cancer axis has greatly broadened the understanding of PDAC biology. While PDAC has long been viewed mainly as a genetically driven malignancy, growing evidence suggests that microbial communities in the gastrointestinal tract and the pancreatic tumor niche can affect tumor initiation, immune suppression, and sensitivity to therapy. In contrast to many epithelial cancers in which microbial influences are largely mediated through local mucosal inflammation, PDAC is defined by intricate interactions among microbial signals, pancreatic immune cells, stromal components, and systemic immune regulation. A recurring mechanistic theme supported by both preclinical and clinical research is that dysbiotic microbial communities can foster an immunosuppressive tumor environment by reshaping innate and adaptive immune pathways. Pushalkar et al. (52) showed that the pancreatic cancer microbiome promotes oncogenesis by triggering immune suppression via selective Toll-like receptor signaling in monocytic populations, which leads to heightened immunosuppressive myeloid activity and diminished T-cell activation. Depletion of the microbiome reversed these immune disturbances, driving M1 macrophage differentiation, strengthening CD4^+^ Th1 responses, boosting CD8^+^ T-cell activation, and enhancing sensitivity to immune checkpoint blockade. Likewise, Riquelme et al. (53) reported that microbial diversity and composition within PDAC tumors correlate with patient outcomes. Long-term survivors displayed distinct intratumoral microbial profiles, and transferring microbiota from different patient groups modified tumor growth and immune infiltration in experimental models. Together, these results indicate that microbial communities may serve not only as passive biomarkers but also as active regulators of tumor–immune interactions. The fungal fraction of the microbiome may also play a role in PDAC progression. Aykut et al. (54) showed that pancreatic tumors display substantial changes in fungal communities, including the movement of gut-derived fungi into pancreatic tissue. The overrepresentation of particular fungal taxa supported tumor development by triggering mannose-binding lectin (MBL)-dependent complement signaling, underscoring that microbial effects are not limited to bacteria and instead reflect wider ecological shifts within the tumor-associated microbiome. Taken together, these findings support a framework in which microbial dysbiosis contributes to PDAC through converging pathways that involve activation of innate immune responses, complement signaling, immune exclusion, and weakened antitumor immunity. Nonetheless, microbial composition differs greatly across geographic region, diet, antibiotic exposure, disease stage, and sequencing approach, which constitutes a major source of variability and makes straightforward translation challenging.

### 6.2 Microbial transformation of naringenin and modulation of host bioavailability

Naringenin is a significant dietary flavanone example in which biological activity is strongly affected by metabolism carried out by intestinal microorganisms. After oral administration, only a portion of intact naringenin enters systemic circulation, since extensive biotransformation takes place within the gastrointestinal tract. Intestinal bacteria are able to alter flavonoids through deglycosylation, hydroxylation, and ring-cleavage reactions, producing metabolites that may display biological activities different from those of the parent compound. Consequently, the biological effects ascribed to naringenin should not be regarded as depending solely on the concentration of the original molecule. Rather, the gut microbiota may act as a metabolic regulator that governs the range, bioavailability, and activity of naringenin-derived metabolites. Research examining the microbial transformation of flavonoids has shown that intestinal bacteria extensively metabolize naringin to yield its aglycone naringenin and other smaller phenolic metabolites (55). This concept carries significant implications for cancer biology, since microbial composition varies substantially across individuals. Differences in microbial enzymatic capacity could help account for variation in flavonoid metabolism and, therefore, for differences in biological responses to naringenin supplementation. Such variability may partly explain why dietary flavonoids often produce encouraging anticancer effects in experimental models, yet yield inconsistent results in clinical contexts. More recent experimental work also indicates that naringenin itself can alter intestinal microbial ecology. In models of metabolic disease, naringenin supplementation alleviated gut microbial dysregulation, increased populations of beneficial bacteria, decreased taxa that may be harmful, and improved intestinal barrier integrity. Together, these findings point to a bidirectional relationship in which the microbiota affect naringenin metabolism, while naringenin, in turn, can reorganize microbial communities. However, direct evidence connecting naringenin-driven gut microbiome reorganization to PDAC progression is still lacking. As a result, the current body of knowledge endorses a mechanistic hypothesis rather than a proven therapeutic route: naringenin may affect PDAC-related biology in part through microbiome-dependent metabolic and immunological mechanisms that must be confirmed in pancreatic cancer models.

### 6.3 Gut-derived microbial metabolites and immune regulation

Beyond microbial composition, the microbiome’s functional outputs including metabolites and microbial-associated molecular patterns, serve as key mediators of host–tumor communication. Microbial metabolites can shape immune cell differentiation, inflammatory signaling, epithelial barrier function, and responses to anticancer therapies. A central mechanistic motif highlighted by cancer immunology research is that microbiota-derived signals can modulate the activity of antigen-presenting cells and T lymphocytes. Viaud et al. (56) showed that intestinal microbiota contributes to the anticancer effectiveness of cyclophosphamide by promoting the generation of immune responses that involve pathogenic Th17 cells and memory Th1 responses. Likewise, Sivan et al. (57) found that commensal Bifidobacterium enhances dendritic cell function and improves CD8^+^ T-cell priming, thereby boosting antitumor immunity and increasing the efficacy of checkpoint blockade. These results offer a conceptual framework for explaining how microbial changes associated with naringenin might affect tumor immunity. If naringenin alters microbial communities or shifts microbial metabolite production, subsequent outcomes could involve changes in antigen presentation, cytokine production, and lymphocyte recruitment. Such pathways are especially important in PDAC, where insufficient immune activation and poor cytotoxic T-cell infiltration pose major therapeutic obstacles. However, the connection between the microbiota and immunity is strongly context dependent. Microbial signals that promote immune activation in one tumor type may have minimal, reduced, or distinct effects in PDAC due to its characteristic desmoplastic microenvironment, plentiful immunosuppressive myeloid populations, and low baseline immunogenicity. Accordingly, findings derived from melanoma or systemic cancer immunotherapy models should be extended with caution.

### 6.4 Gut–pancreas immune communication and therapeutic implications

The gut–pancreas axis constitutes a possible communication network by which changes in intestinal microbial communities may affect pancreatic tumor biology. This relationship may be mediated through multiple interconnected mechanisms, such as microbial migration, circulating metabolites, immune cell trafficking, and systemic inflammatory signaling. In PDAC, available evidence indicates that microbial communities can directly modulate sensitivity to therapy. Routy et al. (58) showed that the composition of the gut microbiome shapes the effectiveness of PD-1–based immunotherapy across epithelial cancers. Antibiotic administration weakened treatment responses, whereas transferring microbiota from responders enhanced the efficacy of checkpoint blockade in experimental settings. A higher abundance of *Akkermansia muciniphila* was linked to improved immune responses, mediated by greater recruitment of immune cells into tumors. Although immunotherapy responses in PDAC remain limited relative to those in other cancers, these results underscore a more general principle: microbial ecosystems can shape treatment sensitivity by altering immune readiness. Accordingly, microbiota-targeting interventions, including dietary compounds such as naringenin, offer an appealing yet still experimental approach to strengthening tumor–immune interactions. From a translational standpoint, naringenin could theoretically affect this axis through several converging mechanisms, including changes in microbial composition, regulation of intestinal barrier integrity, attenuation of inflammatory signaling, and modification of immune-related pathways. Nevertheless, it is unclear whether such effects are adequate to counteract the strongly immunosuppressive PDAC microenvironment. Future research should therefore combine microbiome sequencing, metabolomics, immune profiling, and tumor models to assess whether naringenin-driven microbial alterations lead to functional outcomes in pancreatic cancer. Personalized strategies that account for baseline microbiota composition may also be required, since interindividual microbial variability is likely to affect treatment response.

### 6.5 Critical considerations and future directions

Despite growing interest in microbiota-targeted approaches, several limitations warrant consideration. First, direct evidence linking naringenin supplementation to microbiota-dependent modulation of PDAC is still lacking. At present, the prevailing conclusions rest on two complementary but distinct lines of evidence: investigations demonstrating the involvement of the microbiota in pancreatic cancer, and studies showing that naringenin can modulate the microbiota or immune pathways in other models. Second, microbiome research is constrained by substantial methodological heterogeneity, including differences in sequencing platforms, sample origins, dietary background, antibiotic exposure, and disease stage. These factors hinder meaningful cross-study comparisons and may account for the discrepancies observed in the microbial signatures that have been identified. Third, the biological activity of naringenin is likely influenced not only by microbial composition but also by the production of metabolites, host genetics, intestinal absorption, and the immune environment specific to the tumor. Accordingly, future studies should go beyond taxonomic profiling and instead assess functional microbial outputs, including metabolomics and downstream immune effects (**Table 3**). In particular, targeted metabolomic profiling of microbiota-derived short-chain fatty acids (SCFAs), such as acetate, propionate, and butyrate, together with secondary bile acids, could provide a feasible approach to empirically connect microbiota alterations with host immune modulation (60, 61). These analyses could be integrated with immune profiling of the tumor and intestinal compartments to determine whether microbiota-associated changes in SCFAs or secondary bile acids coincide with alterations in inflammatory signaling, immune-cell composition, or T-cell activity (60, 61). Such an approach would help distinguish correlations in microbial abundance from functional microbiota–host interactions and could clarify whether the immunomodulatory effects proposed for naringenin are mediated, at least in part, through microbiota-derived metabolites.

**Table 3:** Microbiota-dependent mechanisms through which naringenin may influence pancreatic cancer.

Experimental model	Microbiota related mechanism	Main findings	Evidence classification	Potential relevance to pancreatic cancer	Ref
Human and murine pancreatic ductal adenocarcinoma (PDAC) models	Tumor associated microbiome; TLR mediated immune regulation	Increased pancreatic microbiome abundance; microbiome depletion led to decreased MDSCs, increased M1 macrophage differentiation, increased CD4+ Th1 and CD8+ T cell activation	Direct evidence in PDAC (Microbiome context)	Indicates that microbiota can regulate PDAC immune suppression and represents a potential pathway that may be influenced by microbiota modulating compounds such as naringenin	(52)
Human PDAC cohorts; fecal microbiota transplantation mouse models	Intratumoral microbiome diversity and immune microbial interactions	Increased microbial diversity associated with long term survival; microbiota transfer altered tumor growth and immune infiltration	Direct evidence in PDAC (Microbiome context)	Supports the concept that microbial composition may influence PDAC progression and immune phenotype	(53)
Human and murine PDAC models	Gut pancreas fungal translocation; complement activation	Increased intratumoral fungi; Malassezia enrichment; fungal depletion led to decreased tumor progression; MBL/C3 signaling required for oncogenic effect	Direct evidence in PDAC (Microbiome context)	Demonstrates that microbial derived signals beyond bacteria contribute to PDAC pathogenesis and immune regulation	(54)
Mouse tumor models treated with cyclophosphamide	Gut microbiota mediated immune activation	Gut bacteria promoted Th17 and Th1 immune responses required for optimal anticancer activity	Evidence in other cancer models	Provides mechanistic evidence that intestinal microbiota can shape systemic antitumor immunity	(56)
Mouse melanoma models with microbiota manipulation	Commensal bacteria dendritic cell CD8+ T cell axis	Increased Bifidobacterium abundance led to increased dendritic cell function, increased CD8+ T cell priming and improved anti PD-L1 response	Evidence in other cancer models	Supports microbiota dependent regulation of adaptive immunity, a mechanism potentially relevant to PDAC immunomodulation	(57)
Mouse models and cancer patient cohorts receiving PD-1 blockade	Gut microbiome regulation of immune checkpoint efficacy	Antibiotics impaired PD-1 response; Akkermansia muciniphila associated with improved immunotherapy efficacy and increased immune cell recruitment	Evidence in other cancer models	Highlights microbiota as a determinant of therapeutic responsiveness and suggests possible interactions with dietary bioactive compounds	(58)
MCD diet induced NASH mice	Naringenin mediated microbiome and metabolome remodeling	Increased microbiota homeostasis; altered microbial composition; modulation of host metabolome; decreased inflammatory and metabolic abnormalities	Evidence from non-cancer/inflammatory/metabolic models	Provides direct evidence that naringenin can regulate microbiota host interactions, supporting a mechanistic basis for investigating similar pathways in PDAC (Hypothesis-generating evidence)	(59)

CD8^+^ T cells, cytotoxic T lymphocytes; MDSCs, myeloid-derived suppressor cells; MBL, mannose-binding lectin; PDAC, pancreatic ductal adenocarcinoma; PD-1, programmed cell death protein-1; PD-L1, programmed death-ligand 1; TLR, Toll-like receptor.

## 7 Naringenin-mediated strategies to modulate therapeutic resistance in pancreatic cancer

Therapeutic resistance in PDAC is a multifactorial process shaped by tumor-cell intrinsic changes, bidirectional interactions with the microenvironment, metabolic reprogramming, and compromised drug delivery. Even though gemcitabine continues to be a key element of treatment for pancreatic cancer, poor long-term responses are often linked to epithelial–mesenchymal transition (EMT) activation, resistance to apoptosis, enhanced cancer stemness, and the reorganization of signaling pathways that help maintain tumor survival (62–64). Consequently, strategies that can concurrently address multiple mechanisms underlying resistance have received growing attention. Naringenin, a citrus-derived flavanone, has been explored as a possible sensitizer owing to its capacity to modulate oxidative stress, inflammatory signaling, cellular plasticity, and constraints in drug delivery. Nevertheless, the existing body of evidence is largely preclinical, so characterizing naringenin as a resistance-modulating agent should be approached with caution. Current research indicates that naringenin might affect several biological processes implicated in therapeutic resistance, such as EMT-linked adaptation, disruption of redox homeostasis, and constrained bioavailability; however, clinical confirmation remains insufficient (**Table 4**).

**Table 4:** Preclinical evidence for naringenin-mediated therapeutic sensitization and anticancer strategies in pancreatic cancer.

Experimental model	Naringenin based intervention	Main molecular mechanism(s)	Major findings	Evidence classification	Ref
Human pancreatic cancer cell models (*in vitro*)	Naringenin loaded PLGA nanoparticles	Nanoformulation mediated improvement of solubility, stability, and cellular delivery	Naringenin nanoparticles showed sustained release characteristics and greater cytotoxic activity compared with free naringenin	Direct evidence in PDAC	(65)
Human pancreatic cancer cells and xenograft model	Combined naringenin and hesperetin treatment	FAK and p38 signaling inhibition; enhanced caspase-3 activation	Combined flavanone treatment produced stronger inhibition of tumor growth and migration than individual compounds, with reduced toxicity toward normal cells	Direct evidence in PDAC	(66)
SNU-213 human pancreatic cancer cells	Naringenin alone	ROS accumulation led to Prdx-1 suppression, ASK1 activation, and JNK/p38/p53 signaling	Naringenin increased oxidative stress associated apoptotic signaling and reduced pancreatic cancer cell survival	Direct evidence in PDAC	(40)
AsPC-1 and PANC-1 pancreatic cancer cells	Naringenin treatment with gemcitabine exposure	TGF-beta1/Smad3 pathway inhibition; suppression of EMT markers (vimentin, N-cadherin, MMP2/MMP9)	Naringenin reduced migration/invasion and partially restored gemcitabine sensitivity in EMT induced resistant cells	Direct evidence in PDAC	(34)
Multiple human cancer cell lines including PK-1 pancreatic cancer cells; mouse sarcoma model	Naringenin administration	Apoptosis induction and growth inhibition	Naringenin inhibited proliferation of pancreatic cancer cells among several tumor types and showed antitumor activity *in vivo* in a non-pancreatic model	Evidence in other cancer models/Direct evidence in PDAC	(67)
Human pancreatic cancer cells and xenograft model	Citrus derived fermented extract containing naringenin and hesperetin	Activation of MKK3/6-p38 signaling; caspase-3 mediated apoptosis	Citrus flavanone rich extract inhibited pancreatic cancer cell viability, migration, and tumor growth; naringenin and hesperetin were identified as important anticancer components	Direct evidence in PDAC	(68)

Nar, naringenin; Hesp, hesperetin; PLGA, poly(lactic-co-glycolic acid); NPs, nanoparticles; ROS, reactive oxygen species; Prdx-1, peroxiredoxin-1; ASK1, apoptosis signal-regulating kinase 1; JNK, c-Jun N-terminal kinase; p38, p38 mitogen-activated protein kinase; p53, tumor protein p53; EMT, epithelial–mesenchymal transition; TGF-β, transforming growth factor-beta; Smad3, SMAD family member 3; Gem, gemcitabine; FAK, focal adhesion kinase; MKK3/6, mitogen-activated protein kinase kinase 3/6; PK-1, human pancreatic cancer cell line; SNU-213, human pancreatic cancer cell line; AsPC-1, human pancreatic adenocarcinoma cell line; PANC-1, human pancreatic carcinoma cell line; HUVECs, human umbilical vein endothelial cells; fCUP, fermented *Citrus unshiu* peel extract.

### 7.1 EMT-driven plasticity and gemcitabine resistance: targeting phenotypic adaptation

EMT is widely acknowledged as a key process that drives pancreatic cancer progression and contributes to resistance to treatment. During EMT, epithelial tumor cells gain mesenchymal traits, including diminished cell–cell adhesion, greater migratory ability, higher invasive capacity, and modified sensitivity to chemotherapy. In PDAC, EMT is strongly linked to TGF-β pathway activation, extracellular matrix remodeling, and the emergence of a drug-resistant phenotype (69, 70). Notably, EMT should not be viewed as a simple on–off switch; instead, it reflects a dynamic continuum of cellular states that allows tumor cells to adjust to the pressures imposed by therapy.

Direct PDAC evidence:

In direct pancreatic cancer models (AsPC-1, PANC-1, and SNU-213), naringenin has shown clear, cell-intrinsic activity. Specifically, naringenin suppresses TGF-β1/Smad3 signaling to reverse epithelial-to-mesenchymal transition (EMT) and restore gemcitabine sensitivity in AsPC-1/PANC-1 cells, while triggering ROS-dependent ASK1/JNK apoptosis in SNU-213 cells (34, 40).

Supportive non-PDAC evidence:

Complementary studies in non-pancreatic tumor lines further demonstrate that naringenin disrupts cellular redox homeostasis and enhances chemotherapy-induced cell death (Ramos et al., 2008). However, these non-PDAC findings serve strictly as secondary supportive evidence for the direct PDAC mechanisms identified above (71).

The importance of these findings goes beyond blocking invasion, since epithelial–mesenchymal transition is increasingly viewed as a modulator of therapeutic adaptation. By restricting epithelial–mesenchymal transition–related phenotypic plasticity, naringenin may diminish the capacity of pancreatic cancer cells to shift into a drug-tolerant state. Although naringenin-loaded PLGA nanoparticles have demonstrated enhanced cytotoxicity and sustained drug release in pancreatic cancer cell lines *in vitro*, these initial findings must not be interpreted as proof that nanoformulation has resolved naringenin’s systemic bioavailability limitations or established clinical efficacy. Demonstrating therapeutic utility requires systematic progression along a rigorous translational continuum:

- Formulation Feasibility: Physicochemical characterization, encapsulation efficiency, and controlled release kinetics *in vitro*.
- Pharmacokinetic Improvement: Verification of extended plasma half-life, reduced metabolic clearance, and superior oral/systemic exposure *in vivo*.
- Tumor Targeting: Confirmation of preferential drug accumulation within the dense, desmoplastic stroma of pancreatic tumors *in vivo*.
- *In vivo* Efficacy: Demonstration of tumor regression, inhibition of metastasis, and safety in immunocompetent and patient-derived xenograft (PDX) models.
- Clinical Translation: Evaluation of safety, tolerability, and clinical endpoints in human clinical trials.

Currently, naringenin-loaded nanocarriers in PDAC remain restricted to the initial stage of formulation feasibility and *in vitro* cytotoxicity. Comprehensive *in vivo* pharmacokinetic, biodistribution, and therapeutic efficacy studies are strictly required before any potential clinical benefit can be claimed (**Figure 2**).

**Figure 2:**
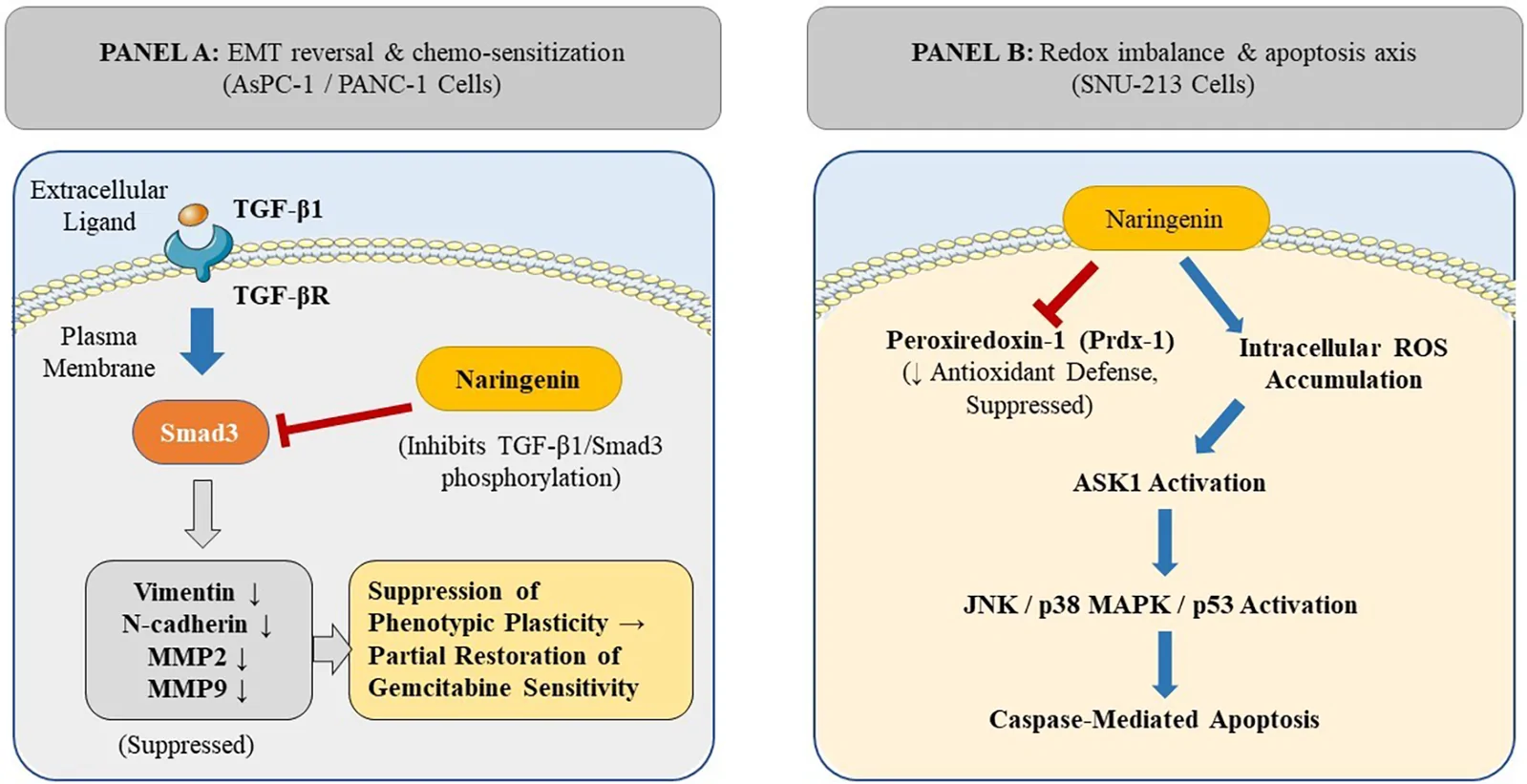
Naringenin modulates pancreatic cancer cell plasticity, gemcitabine chemo-sensitization, and ROS-dependent apoptosis. **(A)** EMT reversal and chemo-sensitization: naringenin suppresses TGF-β1/Smad3 signaling in AsPC-1 and PANC-1 pancreatic cancer cell models. This inhibition downregulates key mesenchymal markers (vimentin, N-cadherin, MMP2, and MMP9), thereby reversing epithelial–mesenchymal transition (EMT)-driven phenotypic plasticity and partially restoring sensitivity to gemcitabine. **(B)** Redox vulnerability and apoptotic cascade: in SNU-213 pancreatic cancer cells, naringenin suppresses peroxiredoxin-1 (Prdx-1) expression while inducing intracellular reactive oxygen species (ROS) accumulation. The resulting redox imbalance activates the ASK1–JNK/p38/p53 signaling pathway, triggering caspase-mediated apoptotic cell death. Data stem from preclinical *in vitro* models; translational efficacy in clinical human pancreatic cancer requires further validation.

### 7.2 Redox vulnerability and apoptosis sensitization in pancreatic cancer cells

Modified regulation of redox states constitutes another key element underlying resistance to pancreatic cancer therapy. PDAC cells commonly sustain an elevated antioxidant capacity that enables them to withstand oxidative stress arising from rapid proliferation, metabolic disturbances, and anticancer treatments. This acquired reliance on antioxidant pathways may expose a therapeutic weakness, such that excessive ROS accumulation can initiate apoptosis or diminish tumor cell survival (72). Naringenin has been shown to interfere with redox homeostasis in pancreatic cancer cells. In SNU-213 pancreatic cancer cells, naringenin increased intracellular ROS accumulation and lowered the expression of peroxiredoxin-1, an antioxidant regulator that contributes to maintaining cellular redox balance. The increase in oxidative stress was linked to activation of apoptosis signal-regulating kinase 1, which was followed by stimulation of JNK, p38 MAPK, and p53 signaling pathways, ultimately driving apoptotic cell death (40). This mechanism may have implications for therapy-induced sensitization, since defective apoptosis signaling is a common characteristic of chemotherapy-resistant pancreatic cancer. Instead of acting only as a direct cytotoxic agent, naringenin could heighten the vulnerability of tumor cells by interfering with survival pathways that enable adaptation to cellular stress. Comparable redox-modulating effects of flavonoids have been suggested as a basis for their anticancer activity, though the extent and direction of ROS regulation are determined by concentration, cellular context, and the antioxidant capacity of each tumor (71). Nonetheless, converting ROS-centered mechanistic concepts into clinical therapeutic strategies necessitates careful evaluation. Overly high systemic oxidative stress could, in principle, influence normal tissues, and achieving selective targeting of tumor-specific redox abnormalities remains a major obstacle. In addition, the concentrations of naringenin applied in experimental studies may surpass levels that can realistically be reached in plasma due to its limited bioavailability. Consequently, although ROS-mediated apoptosis is a biologically credible mechanism for naringenin-driven tumor sensitization, additional pharmacokinetic and *in vivo* investigations are needed.

### 7.3 Improving therapeutic exposure through nanoformulation and flavonoid-based combination strategies

In addition to intrinsic cellular resistance mechanisms, the therapeutic potential of naringenin is also shaped by pharmacological constraints, especially poor aqueous solubility, extensive metabolism, and comparatively low systemic bioavailability. Collectively, these factors pose major obstacles to converting encouraging *in vitro* anticancer activity into clinically significant outcomes. Accordingly, enhancing tumor exposure through advanced delivery systems or rational combination strategies has become an important focus in naringenin-based cancer research. Nanotechnology-enabled formulations have been proposed to address the physicochemical limitations of flavonoids by improving stability, solubility, controlled release, and cellular uptake. In pancreatic cancer models, naringenin-loaded poly(lactic-co-glycolic acid) nanoparticles were designed to improve the delivery performance of naringenin. The optimized nanoparticles exhibited nanoscale dimensions, sustained drug-release behavior, and greater cytotoxic activity than free naringenin *in vitro* (65). These results indicate that formulation strategies could enhance the biological availability of naringenin and may also increase its therapeutic index. Nevertheless, whether higher *in vitro* cytotoxicity translates into better antitumor efficacy *in vivo* is still unclear, since nanoparticle distribution, tumor penetration, immune interactions, and long-term safety must be more thoroughly assessed. Combination regimens offer another approach for addressing therapeutic constraints by simultaneously targeting multiple biological vulnerabilities. Pancreatic cancer is marked by extensive signaling redundancy, implying that blocking a single pathway alone may not be sufficient to achieve sustained responses. Consequently, pairing bioactive compounds with complementary mechanisms may provide a basis for improving anticancer activity. Combined administration of naringenin and hesperetin produced greater inhibition of pancreatic cancer cell proliferation and migration than either agent used independently. Mechanistically, the combination lowered focal adhesion kinase and p38 signaling activity and promoted caspase-3-mediated apoptosis, while exerting only limited effects on normal cell models (66). The potential benefit of flavonoid combinations may stem from their capacity to modulate multiple distinct yet interrelated pathways at the same time, including survival signaling, migration, and regulation of apoptosis. However, these findings should be interpreted with caution, since synergistic effects seen in experimental settings may be highly dependent on dose ratios, length of exposure, and the specific tumor context. Moreover, pharmacological interactions between dietary flavonoids and conventional anticancer agents are still insufficiently understood. Accordingly, future research should prioritize identifying optimal combination strategies, characterizing pharmacokinetic interactions, and determining concentrations that can be realistically achieved in clinical settings.

### 7.4 Potential implications for cancer stemness, targeted therapy, and immunotherapy resistance

Cancer stem cell (CSC) properties, epithelial to mesenchymal transition (EMT), tumor initiating capacity, and the emergence of drug tolerant persister cells are distinct but interconnected phenomena that drive therapeutic failure and minimal residual disease in pancreatic cancer. While direct evidence assessing naringenin against pancreatic CSCs is not yet available, its ability to modulate EMT linked pathways offers a mechanistic basis for future study. Specifically, the inhibition of TGF-β/Smad3 mediated EMT and the partial restoration of gemcitabine sensitivity in pancreatic cancer cells suggest that naringenin affects phenotypic plasticity (34). However, it is critical to emphasize that blocking EMT or reducing cellular plasticity does not equate to the elimination of CSCs. To conclusively support any claims regarding CSCs, future studies must provide dedicated evidence utilizing specific markers such as CD44, CD133, and ALDH1. Furthermore, functional assays including sphere formation, *in vivo* tumor initiation, and lineage tracing studies are strictly required to determine whether naringenin can effectively target drug tolerant persister cells and true CSC populations.

Resistance to targeted therapies and immune checkpoint inhibitors constitutes an additional major obstacle in the management of PDAC. Unlike several other malignancies, pancreatic cancer shows limited responsiveness to immunotherapy, largely because of a highly immunosuppressive tumor microenvironment featuring dense stromal barriers, regulatory immune cell populations, and weakened antitumor T-cell function (4). Naringenin has shown immunomodulatory effects in other experimental settings, including the regulation of inflammatory signaling and immune-cell activity; however, direct evidence that links naringenin to the reversal of pancreatic cancer immunotherapy resistance is presently lacking. Consequently, any possible impact of naringenin on resistance to targeted therapy or immunotherapy should be viewed as hypothesis-generating rather than as an established therapeutic mechanism. Future research should employ immune-competent pancreatic cancer models, patient-derived organoids (PDOs), and combination studies with clinically relevant therapies to assess whether naringenin can affect treatment responsiveness beyond tumor-cell intrinsic processes.

### 7.5 Modulations of CAFs and the extracellular matrix: current gaps and proposed mechanisms

The dense desmoplastic stroma, primarily driven by cancer-associated fibroblasts (CAFs) and extensive extracellular matrix (ECM) remodeling, is a defining hallmark of PDAC. While the abstract of this review touches upon the tumor microenvironment, it is critical to note that direct experimental evidence demonstrating naringenin’s ability to specifically modulate CAFs, pancreatic stellate cell activation, or ECM deposition within PDAC models is currently lacking. Existing literature heavily relies on indirect evidence. For instance, naringenin’s known capacity to inhibit TGF-β signaling, a primary driver of CAF activation, suggests a theoretical mechanism by which naringenin might reduce stromal density. However, this remains a proposed hypothesis. Dedicated *in vivo* studies focusing specifically on naringenin-stellate cell interactions, collagen deposition, and stromal remodeling in PDAC are strictly necessary before definitive claims can be made regarding its efficacy against the desmoplastic barrier.

Future studies could further apply single-cell RNA sequencing (scRNA-seq) to clarify cellular heterogeneity within the PDAC tumor microenvironment and to assess whether naringenin triggers cell-type-specific transcriptional alterations. Specifically, scRNA-seq could differentiate functionally distinct CAF subpopulations, including inflammatory, myofibroblastic, and antigen-presenting CAF states, while concurrently profiling heterogeneous populations of tumor-infiltrating lymphocytes (TILs) and their activation, effector, or dysfunctional states (73, 74). Combining these single-cell transcriptional signatures with measurements of stromal composition, extracellular matrix remodeling, and immune-cell infiltration may help pinpoint cellular populations that respond selectively to naringenin and elucidate possible interactions between CAFs and TILs. These investigations would offer a more high-resolution strategy for evaluating the hypothesized effects of naringenin on the stromal and immune compartments of PDAC, while noting that these effects still require experimental validation.

### 7.6 Translational challenges and future directions

Although preclinical results are promising, several key obstacles must be overcome before naringenin can be regarded as a clinically meaningful adjunct therapy for pancreatic cancer. The initial hurdle is the scarcity of human pharmacological information that specifies what tissue concentrations can realistically be reached. Numerous anticancer effects reported in cell-based studies depend on concentrations that conventional dietary supplementation may not achieve. Consequently, formulation refinement and pharmacokinetic assessment are crucial to establish whether biologically active exposure levels can be attained in living organisms. A further challenge is the inherent complexity of pancreatic cancer biology. PDAC is driven not only by malignant epithelial cells, but also by extensive cross-talk among fibroblasts, immune cells, extracellular matrix constituents, and metabolic signaling networks. Current investigations of naringenin have largely centered on tumor-cell models, which may not fully reflect these multicellular interactions. Future work should therefore test naringenin in more representative systems, including genetically engineered mouse models, organoid platforms, and approaches grounded in the tumor microenvironment.

A complementary future research framework should combine patient-derived organoids with multi-omics profiling to assess whether the proposed molecular effects of naringenin can be reproduced across biologically distinct PDAC contexts. Specifically, organoids generated from tumors that reflect different molecular and clinical phenotypes could be treated with pharmacologically relevant concentrations of naringenin, and then subjected to RNA sequencing and mass spectrometry-based proteomic profiling to evaluate treatment-related changes in gene expression and protein abundance (75, 76). Integrating these datasets with functional drug-response measurements could help separate reproducible molecular effects from adaptations that vary between patients, and it could also reveal candidate biomarkers linked to sensitivity or resistance. This strategy would further offer a way to evaluate whether the pathways suggested in this review such as inflammatory signaling, metabolic reprogramming, oxidative stress, and tumor–stromal interactions, are supported by coordinated changes in transcriptomes and proteomes. Importantly, these experiments should be viewed as a proposed validation approach rather than as proof that is currently established for naringenin in PDAC. Where possible, comparing with matched parental tumor tissue and adding complementary functional assays would enhance the translational relevance of this multi-omics PDO framework.

A further key factor is the heterogeneity of mechanisms underlying resistance to therapy. Although naringenin has demonstrated the capacity to influence epithelial-to-mesenchymal transition, apoptosis, oxidative stress, and constraints related to drug delivery, resistance in pancreatic cancer emerges via several intersecting pathways. Therefore, naringenin is unlikely to serve as a stand-alone therapeutic approach, but it may have stronger translational value when used within rational combination regimens. Taken together, existing findings suggest that naringenin could affect pancreatic cancer therapeutic resistance through multiple converging processes, including inhibition of epithelial-to-mesenchymal transition–linked adaptive responses, promotion of apoptosis via redox modulation, enhancement of drug delivery, and synergistic interaction with other bioactive compounds. However, the available evidence is still largely limited to preclinical research, and carefully designed translational studies are needed to establish whether these mechanisms can be translated into clinically meaningful improvements in pancreatic cancer treatment outcomes.

## 8 Translational opportunities and future perspectives

### 8.1 Biomarker-guided precision oncology

Achieving successful clinical translation of naringenin in pancreatic cancer will depend on moving from a broadly acting phytochemical to a therapeutic strategy informed by biomarkers. PDAC shows marked heterogeneity across patients, with differences that include distinct genomic architectures, transcriptional subtypes, immune characteristics, and treatment vulnerabilities. Accordingly, presuming that all patients would respond in the same way to naringenin-based interventions could restrict its clinical utility. Precision oncology strategies that combine molecular classification with predictive biomarkers may help pinpoint the patient groups most likely to benefit from naringenin-containing therapies (77). A key translational obstacle is determining biomarkers that can forecast responsiveness to naringenin. Extensive genomic and transcriptomic profiling may uncover molecular patterns linked to sensitivity to flavonoid-based treatments, while circulating biomarkers such as circulating tumor DNA, extracellular vesicles, and soluble inflammatory mediators, may enable minimally invasive options for monitoring treatment response. These approaches have grown increasingly important in the management of pancreatic cancer by allowing real-time assessment of tumor progression and treatment resistance (78). Future research should test whether baseline molecular features, such as particular tumor phenotypes or microenvironmental conditions, can forecast improved responses to naringenin administered alone or alongside standard therapies. In addition, biomarker-guided clinical trials may offer a more effective structure for evaluating naringenin than conventional unselected trial designs. Adaptive enrichment strategies where participants are enrolled according to prespecified biological criteria, could increase the likelihood of identifying therapeutic effects and lessen heterogeneity across clinical trials. Comparable precision medicine strategies have already begun reshaping the development of targeted anticancer therapies in pancreatic cancer by pairing specific molecular alterations with tailored treatment choices (79). Overall, combining biomarker discovery with naringenin investigation is an essential step toward the personalized use of this compound in PDAC. Rather than considering naringenin as a universal anticancer agent, future clinical development should focus on defining which patients, disease contexts, and therapeutic combinations are most suitable for its application.

### 8.2 Nanomedicine and targeted delivery

Although naringenin has shown promising anticancer activity in experimental models, its clinical application is constrained by multiple pharmacological drawbacks, such as low aqueous solubility, reduced stability in the gastrointestinal tract, rapid metabolism, and inadequate systemic bioavailability. Nanomedicine-based delivery platforms may offer a useful approach to address these limitations by strengthening naringenin stability, increasing its accumulation in tumors, and supporting controlled drug release at the tumor site. Recent progress in nanoparticle engineering has indicated that encapsulating bioactive compounds can markedly alter their pharmacokinetic behavior and improve therapeutic efficacy relative to free compounds (80). A range of nanocarrier systems including polymeric nanoparticles, lipid-based nanoparticles, nanoemulsions, and targeted nanoconjugates, has been examined for delivering poorly soluble phytochemicals. Such platforms can promote passive tumor targeting via enhanced permeability and retention-driven accumulation, and further surface modification with tumor-specific ligands could enhance selective delivery to pancreatic cancer cells. Importantly, pancreatic tumors are a particularly difficult target due to their dense extracellular matrix, inadequate vascular perfusion, and markedly heterogeneous stromal composition. Accordingly, next-generation naringenin delivery systems should be developed not only to elevate drug accumulation within tumor cells but also to enhance transport through the intricate pancreatic tumor microenvironment (81). Combination nanotechnology strategies may further expand opportunities to maximize naringenin’s therapeutic effectiveness. For instance, co-delivery platforms that pair naringenin with chemotherapeutic agents, immune-modulating molecules, or other phytochemicals could allow coordinated regulation of multiple therapeutic targets while mitigating systemic toxicity. In addition, stimuli-responsive nanoparticles that are triggered by tumor-associated features including acidic pH, oxidative stress, or enzymatic activity, may enable more controlled and accurate spatially and temporally regulated drug release (82). Nevertheless, multiple challenges must be resolved before naringenin-based nanomedicine can proceed to clinical assessment. These challenges include large-scale manufacturing, reproducibility, long-term safety evaluation, biodistribution analysis, and regulatory approval. Future studies should prioritize clinically relevant delivery platforms with validated pharmacokinetic profiles and evaluate whether improved delivery translates into meaningful therapeutic benefits in advanced pancreatic cancer models.

### 8.3 Multi-omics and systems biology

The biological complexity of pancreatic cancer, together with the pleiotropic actions of naringenin, underscores the need for systems-level strategies to assess its translational potential. Traditional single-pathway studies yield important mechanistic understanding; however, they may not fully reflect the evolving interactions among tumor genetics, cellular phenotypes, immune populations, stromal constituents, and metabolic networks that shape therapeutic outcomes. Multi-omics technologies, which integrate genomics, transcriptomics, proteomics, metabolomics, and epigenomics, provide a broad framework for describing these linked processes and for determining the biological settings in which naringenin could deliver the greatest therapeutic advantage (83). In subsequent research, multi-omics profiling could be used to uncover molecular signatures linked to naringenin responsiveness. For instance, combining transcriptomic and proteomic analyses may identify coordinated shifts in tumor-associated pathways, whereas metabolomic methods could test whether naringenin creates particular metabolic vulnerabilities in pancreatic cancer cells. Moreover, spatial omics technologies offer a developing opportunity to examine how naringenin affects interactions between malignant cells and adjacent stromal or immune compartments while maintaining spatial information within the tumor architecture (84). These methods may be especially useful in PDAC, in which treatment resistance is determined in large part by spatial heterogeneity and by intricate tumor–microenvironment interactions. Systems biology approaches may also support the discovery of rational combination regimens. Computational modeling and network-based analyses can integrate multi-tiered biological datasets to forecast synergistic interactions between naringenin and established treatments, including chemotherapy, targeted therapies, or immunotherapeutic strategies. Instead of assessing naringenin as a stand-alone compound, these approaches may embed it in a wider therapeutic network and delineate disease contexts in which its biological effects are most pertinent (85). Even so, putting multi-omics strategies into practice calls for careful standardization, access to large patient cohorts, and validation through functional models. Variations in sample processing methods, analytical platforms, and computational pipelines continue to pose significant obstacles for clinical translation. Accordingly, future work should integrate multi-omics discovery with experimental validation and prospective clinical studies to define reliable biomarkers and mechanistic frameworks that underpin personalized naringenin-based interventions.

### 8.4 PDOs and personalized medicine

The creation of patient-derived models is an important advance toward enhancing the clinical relevance of naringenin research in pancreatic cancer. While standard two-dimensional cell cultures and animal models have yielded crucial insights into the biological effects of naringenin, they frequently cannot capture the complex heterogeneity, three-dimensional structure, and patient-specific traits that characterize pancreatic tumors. PDOs, established from tumor tissues while retaining key genetic and phenotypic characteristics of the original cancer, offer a platform that is more representative of clinical reality for assessing therapeutic responses and individual variation (86). PDO-based models provide multiple benefits for studying naringenin-based interventions. First, they enable direct measurement of between-patient differences in drug sensitivity, which may facilitate the identification of individuals who are more likely to benefit from naringenin-containing treatment regimens. Second, organoids can be applied to test combination strategies, including potential interactions between naringenin and standard-of-care therapies, targeted agents, or emerging immunotherapeutic approaches. Importantly, drug screening based on PDOs has shown the capacity to capture patient-specific treatment responses and may support personalized treatment selection in gastrointestinal cancers (75). Combining PDOs with advanced technologies, including single-cell sequencing, spatial profiling, and functional drug screening, may further increase their translational value. Together, these platforms could allow investigators to examine how tumor heterogeneity, stromal composition, and treatment-driven adaptations affect naringenin responsiveness. In addition, adding immune elements via patient-derived tumor organoid–immune co-culture systems could open further avenues for assessing the interplay between naringenin and antitumor immune responses. Despite these benefits, several limitations need to be addressed before PDO-guided naringenin development can be integrated into clinical workflows. Organoid generation is still technically challenging, success rates differ across patients, and many models fail to fully recapitulate tumor vasculature and the immune ecosystem. In addition, standardized protocols are needed for culture conditions, drug exposure, and response assessment (87). Future studies that combine PDO platforms with multi-omics characterization and prospective clinical validation could support the establishment of a personalized framework to identify which patients with pancreatic cancer are most likely to respond to naringenin-based therapeutic approaches.

### 8.5 Current limitations and future clinical trials

Despite the fact that a growing body of preclinical evidence indicates the therapeutic promise of naringenin in pancreatic cancer, several key limitations currently hinder its progression to clinical use. The majority of existing studies have been conducted *in vitro* systems or animal models, whereas convincing clinical evidence concerning effectiveness, safety, pharmacokinetics, and the most appropriate dosing in patients with pancreatic cancer is still lacking. A central obstacle is establishing whether the concentrations of naringenin needed to generate anticancer activity in experimental models can be reached and sustained in human tissues after oral administration. Variability in absorption, metabolism, bioavailability, and tissue distribution are therefore essential factors that must be rigorously and systematically assessed prior to clinical implementation (19). Another limitation is the absence of standardized formulations and treatment protocols. Research on naringenin often uses different experimental models, doses, administration routes, and outcome measures, which complicates direct comparisons. Future clinical trials should clarify pharmacokinetic and pharmacodynamic relationships, specify biologically effective doses, and determine whether optimized formulations such as nanoparticle-based delivery systems, can enhance therapeutic exposure. Moreover, because pancreatic cancer treatment is increasingly based on multimodal approaches, clinical studies should examine naringenin mainly as a complementary agent used alongside established therapies, rather than as a substitute for current standards of care (88). Future studies should also use biomarker-guided designs to determine which patient groups are most likely to benefit from naringenin-based interventions. In early-phase research, emphasis should be placed on safety, tolerability, pharmacological features, and biological response indicators before assessing clinical efficacy. Adaptive trial designs that combine molecular profiling, organoid-based drug sensitivity testing, and longitudinal biomarker surveillance may hasten the identification of responsive subgroups (89). Importantly, potential challenges connected to regulatory approval, manufacturing quality, consistency of formulation, and long-term safety need to be addressed. Natural compounds often face translational obstacles because biological activity demonstrated in experimental settings does not necessarily result in clinically meaningful effects. Consequently, well-designed randomized clinical trials with rigorous endpoints are required to assess whether naringenin can become a scientifically validated element of pancreatic cancer management (**Table 5**).

**Table 5:** Knowledge gaps and future research priorities for translating naringenin into pancreatic cancer therapy.

Research area	Current evidence	Major limitations	Future directions
Biomarker-guided precision oncology	Naringenin regulates multiple pathways involved in tumor growth, inflammation, immunity, and metabolism.	No validated biomarkers exist to predict patient responsiveness.	Identify predictive signatures using genomic, transcriptomic, immune, and circulating biomarkers.
Nanomedicine and targeted delivery	Nanoformulations may improve naringenin solubility, stability, bioavailability, and tumor targeting.	Limited pharmacokinetic data, formulation variability, and lack of clinical validation.	Develop targeted and stimuli-responsive delivery systems and evaluate combination nanotherapies.
Multi-omics and systems biology	Omics approaches can define molecular responses and identify therapeutic vulnerabilities.	Few integrated multi-omics studies and limited functional validation are available.	Combine genomics, transcriptomics, proteomics, metabolomics, and spatial analyses to establish predictive models.
Patient-derived organoids and personalized medicine	Organoids preserve patient-specific tumor features and enable individualized drug testing.	Incomplete representation of immune, vascular, and stromal components limits clinical translation.	Integrate organoids with single-cell analysis and functional screening for personalized therapy selection.
Clinical translation and trials	Preclinical evidence supports anticancer and immunomodulatory effects of naringenin.	Lack of human trials, optimal dosing, safety, and bioavailability data.	Conduct early-phase clinical trials focusing on pharmacokinetics, safety, biomarkers, and therapeutic efficacy.
Combination therapy optimization	Naringenin may enhance responses to chemotherapy and other anticancer strategies.	Optimal combinations, doses, and treatment schedules remain unclear.	Perform systematic combination screening with chemotherapy, targeted therapy, and immunotherapy.
Microbiota–host interactions	Gut microbiota may influence naringenin metabolism and biological activity.	Specific microbial mediators and clinical relevance remain unknown.	Integrate microbiome and metabolomic approaches to identify microbiota-based predictors of response.

### 8.6 Summary of evidence strength

To provide a clear perspective on the current translational status of naringenin, we have categorized the discussed biological mechanisms based on their level of experimental validation. **Table 6** outlines the strength of the evidence, distinguishing between effects directly demonstrated in pancreatic cancer models, indirect mechanisms observed in other tumor types or inflammatory conditions, and hypothetical pathways that require dedicated future investigation. This stratification highlights the critical need to transition from broad mechanistic hypotheses to targeted, context specific validation in pancreatic ductal adenocarcinoma.

**Table 6:** Evidence strength of naringenin mediated mechanisms relevant to pancreatic cancer.

Evidence category	Biological mechanism	Current level of validation
Demonstrated in PDAC	Inhibition of PI3K/AKT/mTOR and MAPK/ERK signaling	Validated *in vitro* (PANC-1, AsPC-1 cells)
Demonstrated in PDAC	Reversal of epithelial to mesenchymal transition via TGF-beta/Smad3 inhibition	Validated *in vitro* (AsPC-1, PANC-1 cells)
Demonstrated in PDAC	Induction of ROS dependent apoptosis via ASK1 signaling	Validated *in vitro* (SNU-213 cells)
Demonstrated in PDAC	Improved cellular delivery and cytotoxicity via PLGA nanoparticles	Validated *in vitro*
Indirect Evidence	Reprogramming of macrophage polarization and CD8+ T cell recruitment	Demonstrated in oral and hepatocellular carcinoma models
Indirect Evidence	Modulation of gut microbiota and host metabolome	Demonstrated in metabolic and inflammatory disease models
Indirect Evidence	Degradation of VEGFA/c-Met and reduction of PD-L1 expression	Demonstrated in hepatocellular carcinoma models
Hypothetical/Proposed	Direct modulation of cancer associated fibroblasts and desmoplastic stroma	Requires dedicated *in vivo* PDAC validation
Hypothetical/Proposed	Reversal of PDAC specific immune exclusion and immunotherapy resistance	Requires immune competent PDAC models
Hypothetical/Proposed	Targeting of pancreatic cancer stem cells and persister cell populations	Requires lineage tracing and *in vivo* initiation assays
Hypothetical/Proposed	Microbiota dependent modulation of the gut pancreas immune axis	Requires targeted microbiome depletion and transfer in PDAC models

## 9 Clinical translation, pharmacokinetics, and achievable concentrations

Despite promising preclinical results, the translation of naringenin into clinical practice for pancreatic ductal adenocarcinoma (PDAC) is significantly hindered by its pharmacokinetic limitations and a scarcity of clinical evidence. Naringenin exhibits low oral bioavailability in humans, primarily due to poor aqueous solubility and extensive first-pass phase II metabolism, notably glucuronidation and sulfation. Clinical pharmacokinetic studies reveal a significant gap between the high concentrations frequently employed in *in vitro* cell culture models (often ranging from 10 to 200 µM) and the much lower concentrations achievable in human plasma. For instance, a single oral dose of 135 mg of pure naringenin yields a maximum plasma concentration (Cmax) of roughly 7.39 µM, while dietary consumption from food sources typically results in sub-micromolar plasma levels (<1 µM). Furthermore, naringenin is rapidly cleared from systemic circulation, presenting a brief half-life of approximately 2 to 3 hours. Crucially, while clinical trials have investigated naringenin’s safety and its potential in metabolic diseases, there remains a stark absence of specific clinical trials evaluating its efficacy in human cancer, including PDAC. Future clinical translation will heavily depend on the development of novel delivery systems, such as nanoparticle formulations, designed to overcome these bioavailability barriers and achieve sustained, therapeutically relevant concentrations within the dense pancreatic tumor microenvironment.

A crucial next step for establishing the translational relevance of naringenin is to combine formal pharmacokinetic–pharmacodynamic (PK–PD) mathematical modeling with orthotopic pancreatic cancer studies. By using such models, researchers can quantitatively connect administered dose, systemic exposure, time-varying drug concentrations, and tumor-growth dynamics, which enables estimation of exposure–response relationships rather than drawing inferences from single concentration measurements (90, 91). Specifically, future work should assess whether the single-digit micromolar plasma concentrations attainable after oral dosing with naringenin are sufficient to sustain a biologically relevant tumor-suppression threshold over time, while accounting for rapid systemic clearance and the possibility of restricted tumor penetration. Linking plasma, and when feasible intratumoral, concentration–time profiles with pharmacodynamic endpoints such as tumor growth inhibition, apoptosis, proliferation, and pertinent molecular biomarkers would enable estimation of exposure thresholds that correspond to antitumor activity. Implementing these models in orthotopic PDAC models could further separate transient peak exposure from sustained pharmacologically active exposure and support identification of the dose, schedule, and formulation needed to obtain reproducible tumor suppression. This quantitative PK–PD framework should therefore be viewed as an important future avenue for determining whether the experimentally observed anticancer effects of naringenin can be achieved at clinically realistic exposures.

## 10 Conclusion

Pancreatic cancer is marked by a highly complex biological environment in which malignant epithelial cells are present alongside a dense stromal compartment, impaired immune responses, metabolic reprogramming, and continually evolving interactions with the host microbiota. Together, these linked processes drive tumor growth, metastatic spread, and resistance to both established and newly developed therapies. Instead of functioning through a single molecular target, naringenin seems to modulate multiple biological pathways that are pertinent to this complex disease network, such as inflammatory signaling, oxidative stress, mitochondrial activity, cellular metabolism, extracellular matrix remodeling, and immune control. Taken together, naringenin represents a biologically plausible but experimentally and clinically unvalidated adjunctive strategy for pancreatic cancer. Its therapeutic potential remains dependent on establishing pharmacologically achievable exposure, reproducible efficacy in clinically relevant PDAC models, safety, and ultimately benefit in adequately designed clinical trials. Nonetheless, the current body of evidence is still largely limited to preclinical research. Most mechanistic insights have been derived from cultured cell lines or animal models, and relatively few investigations have assessed whether these results produce clinically relevant advantages for individuals with pancreatic cancer. Moreover, differences among experimental models, dosing schedules, formulation approaches, and measured endpoints make straightforward comparisons across studies difficult. Key obstacles including limited oral bioavailability, pharmacokinetic variability, and the lack of standardized biomarkers for treatment response, also need to be addressed before clinical application can be evaluated. Future research should shift from stand-alone mechanistic experiments to coordinated studies that integrate molecular profiling, functional immunology, and clinically relevant disease models. Particular emphasis should be placed on clarifying how naringenin affects tumor immune–stromal interactions, immunometabolic reprogramming, microbiota–host signaling, and therapeutic resistance across diverse patient populations. Use of single-cell and spatial multi-omics methods, PDOs, and biomarker-guided translational research may yield important insights into the biological settings in which naringenin is most likely to be effective. Ultimately, well-designed clinical trials assessing optimized formulations and combination regimens will be necessary to establish its therapeutic value. Continued investigation along these directions may help clarify whether naringenin can contribute to more effective and personalized treatment strategies for pancreatic cancer.
